# Substance P Antagonism as a Novel Therapeutic Option to Enhance Efficacy of Cisplatin in Triple Negative Breast Cancer and Protect PC12 Cells against Cisplatin-Induced Oxidative Stress and Apoptosis

**DOI:** 10.3390/cancers13153871

**Published:** 2021-07-31

**Authors:** Emma Rodriguez, Guangsheng Pei, Sang T. Kim, Alexis German, Prema Robinson

**Affiliations:** 1 Department of Infectious Diseases, Infection Control and Employee Health, The University of Texas MD Anderson Cancer Center, Houston, TX 77030, USA; EERodriguez1@mdanderson.org; 2 Center for Precision Health, School of Biomedical Infomatics, The University of Texas Health Science Center at Houston, Houston, TX 77030, USA; guangheng.pei@uth.tmc.edu; 3 Department of General Internal Medicine, Division of Internal Medicine, The University of Texas MD Anderson Cancer Center, Houston, TX 77030, USA; STKim@mdanderson.org; 4 University of Houston, Houston, TX 77004, USA; abgerman@uh.edu

**Keywords:** Substance P, triple negative breast cancer, cisplatin

## Abstract

**Simple Summary:**

Although cisplatin is very effective as a treatment strategy in triple-negative breast cancer (TNBC), it has unwarranted outcomes owing to recurrence, chemoresistance and neurotoxicity. In the current studies we determined a novel therapeutic option that enhances the efficacy of cisplatin and at the same time protects neuronal cells from cisplatin induced toxicity.

**Abstract:**

Although cisplatin is very effective as a treatment strategy in triple-negative breast cancer (TNBC), it has unwarranted outcomes owing to recurrence, chemoresistance and neurotoxicity. There is critically important to find new, effective and safe therapeutics for TNBC. We determined if SP-receptor antagonism in combination with cisplatin may serve as a novel, more efficacious and safer therapeutic option than existing therapies for TNBC. We used a neuronal cell line (PC12) and two TNBC cell lines (Sum 185 and Sum 159) for these studies. We determined that the levels of cells expressing the high-affinity SP-receptor (neurokinin 1 receptor (NK1R)), as determined by flow-cytometry was significantly elevated in response to cisplatin in all three cells. We determined that treatment with aprepitant, an SP-receptor antagonist decreased cisplatin-induced, loss of viability (studied by MTT assay), production of reactive oxygen species (by DCFDA assay) and apoptosis (by flow-cytometry) in PC12 cells while it was increased in the two TNBC cells. Furthermore, we demonstrated that important genes associated with metastases, inflammation, chemoresistance and cell cycle progression are attenuated by SP-receptor antagonism in the TNBC cell line, Sum 185. These studies implicate that SP-receptor antagonism in combination with cisplatin may possibly serve as a novel, more efficacious and safer therapeutic option than existing therapies for TNBC.

## 1. Introduction

Breast cancer is amongst the foremost cause of cancer-related mortality in women [1]. Triple negative breast cancer (TNBC) is more prevalent in young women and is characterized by aggressive clinical characteristics, the early peak of distant recurrences at 3 years after diagnosis, poor prognosis and high mortality rate within the first 5 years [2,3]. Cisplatin forms the basis of chemotherapy regimens for many malignancies, including TNBC, ovarian and cervical cancers, prostate and testicular cancers, bladder cancer, head and neck cancer, lung cancer and non-Hodgkin’s lymphoma [4,5,6,7]. It mediates its effects by crosslinking with the purine bases on the DNA, thus leading to interference with DNA repair leading to DNA damage and ensuing apoptosis of tumor cells. Though cisplatin is highly effective as first-line therapy in TNBC, it can lead to chemoresistance and unwanted dire manifestations such as peripheral neuropathy, inhibited immune responses to infections, severe kidney damage, hearing loss, allergic reactions, hemorrhage, and gastrointestinal disorders [8,9]. Novel therapeutic combinations are needed to increase the efficacy of cisplatin and at the same time ablate or reduce cisplatin-induced toxicity and chemoresistance.

Substance P (SP), a neuropeptide and pain transmitter acting via its high-affinity receptor, neurokinin1 receptor (NK-1R), has shown to be mitogenic for human cancer cells in vitro [10,11,12,13]. NK1R is overexpressed in TNBC cells [14] and many other cancers, such as head and neck cancer, glioma, astrocytoma, retinoblastoma, ganglioneuroblastoma, leukemia, neuroblastoma and carcinomas (pancreatic, larynx, gastric, colon, medullary thyroid) [13,15]. In vitro studies have shown SP to prevent apoptosis of tumor cells and induce tumor cell migration [10,14,16,17,18,19,20]. Substance P (SP) is known to trigger inflammatory responses, stimulate the production of reactive oxygen species (ROS) [21,22,23]. Our prior studies demonstrate that SP receptor antagonism increases the efficacy of doxorubicin in TNBC cell lines while protecting cardiomyocytes from doxorubicin-induced oxidative stress, apoptosis and chemotherapy-induced death [24]. In the current studies, we will determine if SP receptor antagonism will increase the efficacy of cisplatin, another chemotherapeutic drug in TNBC cell lines. We will also determine if SP receptor antagonism will protect PC12, a rat neuronal cell line, from cisplatin-induced oxidative stress and apoptosis.

In the current studies, we quantitated the levels of SP and NK1R in two TNBC cell lines and in PC12 cells in the presence and absence of cisplatin. We also determined if the levels of apoptosis and ROS will be significantly altered in response to cisplatin treatment. Furthermore, we determined if treatment with an SP receptor antagonist, aprepitant (that is widely used to attenuate chemotherapy-associated nausea), would reverse these cisplatin-induced alterations. Most importantly, we determined if aprepitant would enhance the efficacy of cisplatin in TNBC cells while protecting PC12 cells from cisplatin-induced oxidative stress and apoptosis. The studies in this manuscript will determine if SP receptor antagonism in combination with cisplatin may serve as a novel, more efficacious and safer therapeutic option than existing therapies for TNBC.

## 2. Results

### 2.1. Cisplatin Increases NK1R Levels in Both PC12 Neuronal Cells and TNBC Cells

**Figure 1:**
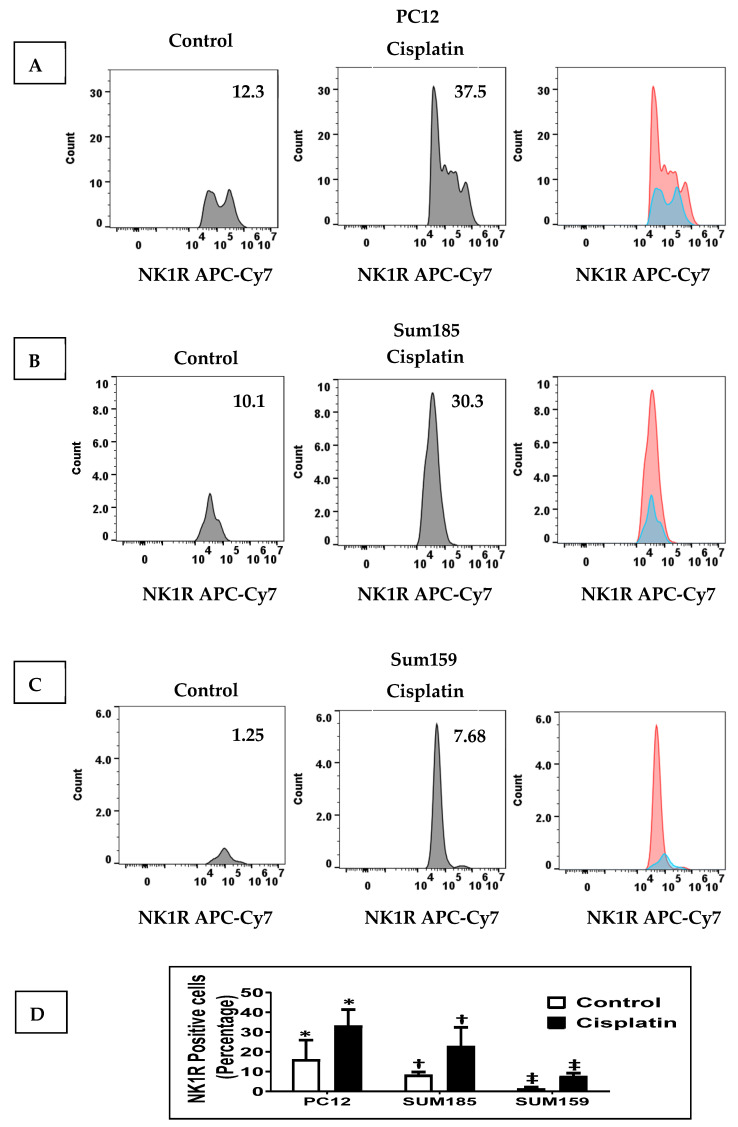
Cisplatin increases NK1R Levels in both PC12 neuronal cells and TNBC Cells. NK1R positive cells as determined by flow cytometry, in cisplatin-treated versus untreated PC12 cells, Sum 185 and Sum 159 cells (**A**–**C**), gate and numbers represent NK1R positive population within live cells. Levels of NK1R positive cells in all three cells (**D**) (*, †, ‡, *p* ≤ 0.05, Student’s *t*-test, *n* = 2).

We determined whether cisplatin treatment increased NK1R levels in PC12 cells and in Sum 185 and Sum 159 TNBC cells. We treated PC12 cells, Sum 185 and Sum 159, with their respective IC_50_ dose of cisplatin for 48 h and then determined SP levels by flow cytometry. We determined the percentage of NK1R positive cells in all three cells in response to cisplatin was significantly higher than the untreated cells. The percentage of NK1R positive cells in the absence of cisplatin, respectively, for PC12, Sum 185 and Sum 159 cells was 16.07% ± 9.85%, 8.27% ± 1.56% and 1.41% ± 0.74%. Cisplatin treatment led to a significant increase in the percentage of NK1R positive cells to 33.1% ± 8.29%, 22.87% ± 9.53% and 7.7% ± 1.56%, respectively, for PC12, Sum 185 and Sum 159 cells. (Figure 1, *p* ≤ 0.05, Students *t*-test, *n* = 2, for all cells).

### 2.2. NK1R Antagonism Protects Rat Neuronal PC12 Cells from Loss of Viability Induced by Cisplatin

**Figure 2:**
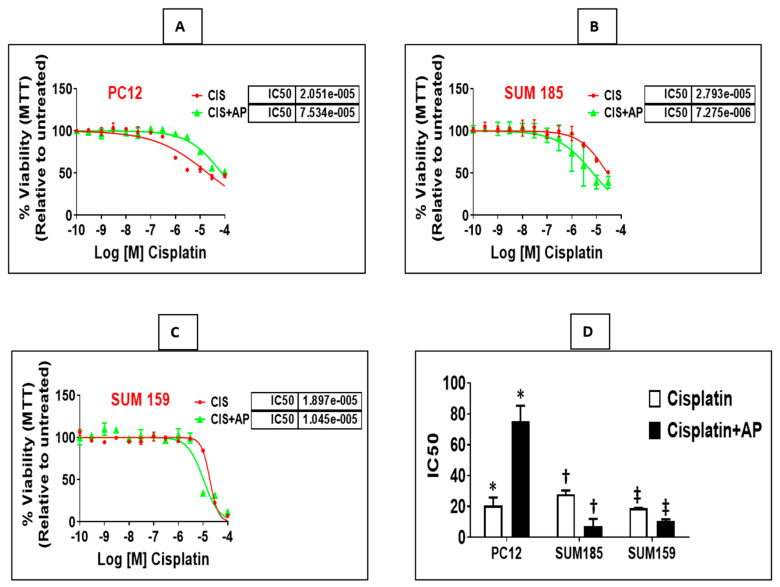
NK1R Antagonism protects rat neuronal PC12 cells while causing enhancement of cisplatin-induced loss of viability in TNBC cells. Loss of viability induced by cisplatin in the presence and absence of aprepitant in rat neuronal PC12 cells, Sum 185 and Sum 159 cells (**A**–**C**) and IC_50_ levels of cisplatin in the presence and absence of aprepitant in PC12 and TNBC cells (**D**) (*, †, ‡, *p* ≤ 0.05, Student’s *t*-test, *n* = 2–3).

We determined if treatment with aprepitant, an NK1R antagonist prevents cisplatin-induced reduction in neuronal viability. PC12 cells were plated in a 96-well plate (3000 cells/well). Twenty-four hours later, we treated triplicate wells with concentrations of cisplatin (ranging from 0.003 μm to 100 μm) in the presence and absence of aprepitant (10 μm, treated 2 h before cisplatin treatment). Moreover, we included control wells consisting of similar concentrations of vehicle (DMSO) that were used in the reconstitution of aprepitant (0.1% DMSO in water), media alone or with aprepitant alone. Each experiment was performed at two different times, and results are represented as mean ± SEM. We elucidated that aprepitant protected PC12 cells from cisplatin-induced loss of viability. The mean inhibitory concentration (IC_50_) of cisplatin was 20.51 μm ± 5.28 μm; the presence of aprepitant with cisplatin led to a 3.6-fold increase in the IC_50_ levels to 75.34 μm ± 10.06 μm (Figure 2A,D; *p* = 0.01, ANOVA, *n* = 2).

### 2.3. NK1R Antagonism Enhances Efficacy of Cisplatin in Two Triple Negative Breast Cancer (TNBC) Cells

We determined if treatment with aprepitant enhances cisplatin-induced reduction in viability of TNBC cells. Sum 185 and Sum 159, human TNBC cells, were plated in a 96-well plate (3000 cells/well). Twenty-four hours later, we treated triplicate wells with concentrations of cisplatin (0.003 μm to 100 μm) in the presence and absence of aprepitant (10 μm, treated 2 h before cisplatin treatment). Moreover, we included control wells consisting of treatment with similar concentrations of vehicle (DMSO) used for reconstitution of aprepitant (0.1% DMSO in water), media alone or wells treated with aprepitant alone. All experiments were performed at two different times, and results are represented as mean ± SEM. We determined that aprepitant enhanced cisplatin-induced loss of viability of both TNBC cells. The mean inhibitory concentration (IC_50_) of cisplatin for Sum 185 was 27.93 μm ± 2.43 μm; the presence of aprepitant with cisplatin led to a 3.84-fold decrease in the IC_50_ levels to 7.27 μm ± 4.67 μm (Figure 2B,D; *p* = 0.001, ANOVA, *n* = 3). The mean inhibitory concentration (IC_50_) of cisplatin for Sum 159 was 18.97 μm ± 0.19 μm; the presence of aprepitant with cisplatin led to a 1.8-fold decrease in the IC_50_ levels to 10.45 μm ± 1.27 μm (Figure 2C,D; *p* ≤ 0.05, ANOVA, *n* = 2).

### 2.4. NK1R Antagonism Attenuates ROS Production Induced by Cisplatin in PC12 Cells

**Figure 3:**
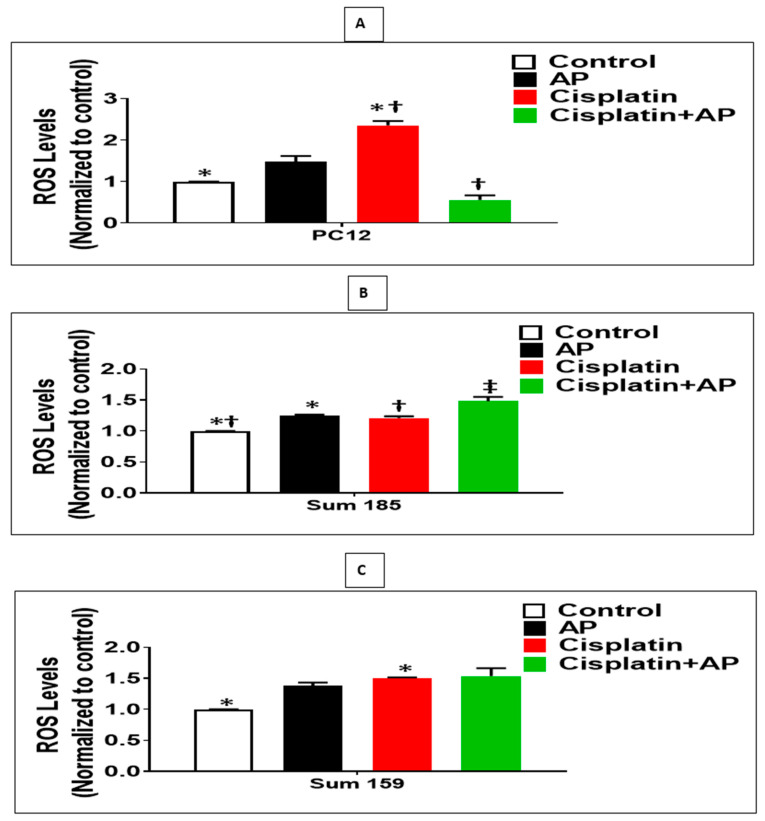
NK1R Antagonism decreased cisplatin-induced ROS in rat neuronal PC12 cells while causing increased cisplatin-induced ROS levels in TNBC cells. Effect of NK1R antagonism on ROS Production induced by cisplatin in PC12 cells and TNBC cells. Levels of ROS as determined by the DCFDA assay, in untreated (media control) or treated with aprepitant or cisplatin alone or cisplatin + aprepitant, in PC12 (**A**), Sum 185 (**B**,**C**), Sum 159 cells (*, †, ‡, *p* ≤ 0.05, ANOVA, *n* = 2). Only statistical comparisons between control vs. aprepitant (AP) or Cis and Cis vs. Cis + AP are shown.

We determined whether the protective effects of SP antagonism on the cisplatin-induced loss of viability and prevention of apoptosis of PC12 cells were accompanied by decreased ROS levels. We determined the levels of cisplatin-induced ROS production in the media control group, the aprepitant alone group, and in the cisplatin group in the presence and absence of aprepitant in PC12 cells. The levels of ROS were normalized to that of the media control group. There was no significant difference in ROS levels between the media control and the aprepitant treated groups (Figure 3A; *p* > 0.05, ANOVA, *n* = 2). The percentage increase in ROS levels in response to cisplatin was significantly higher compared to media control (136% ± 5.3%; Figure 3A; *p* < 0.05, ANOVA, *n* = 2). Most importantly, the percentage increase in ROS levels in response to cisplatin-treated cells in the absence of aprepitant was significantly higher than the cisplatin-treated cells in the presence of aprepitant (79% ± 18.2%; Figure 3A; *p* ≤ 0.05, ANOVA, *n* = 2).

### 2.5. Effect of NK1R Antagonism on ROS Production Induced by Cisplatin in TNBC Cells

**Figure 4:**
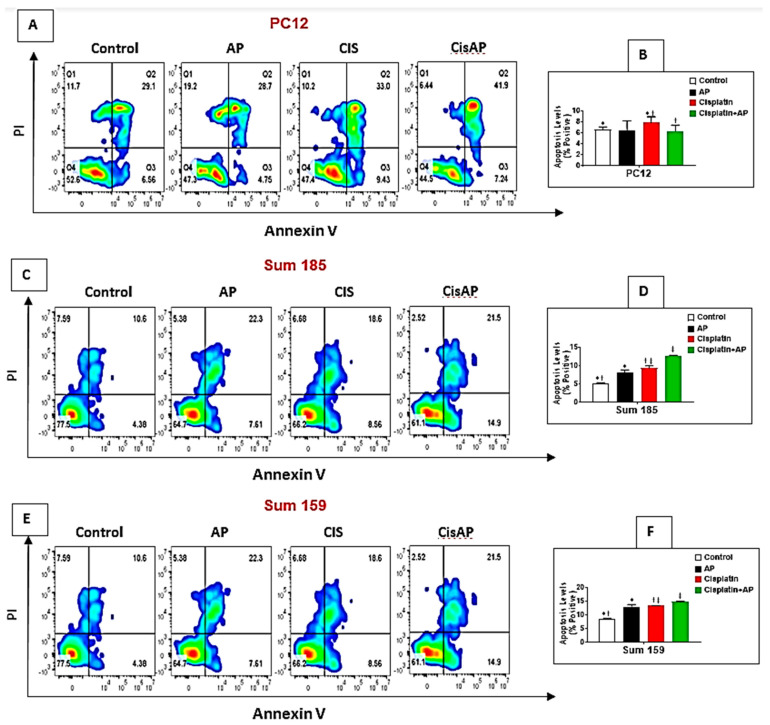
NK1R Antagonism protects PC12 cells while causing enhancement of cisplatin-induced apoptosis in TNBC cells. PC12, Sum 185 and Sum 159 TNBC cells were untreated (media control) or treated with aprepitant or cisplatin alone or cisplatin + aprepitant, stained with Annexin V-FITC/PI and cell apoptosis analyzed by flow cytometry (**A**,**C**,**E**). Quadrant gating was made based on unstained controls. Q2 demonstrates necrotic cells, Q3 demonstrates apoptotic cells, and Q4 demonstrates viable cells. Levels of apoptosis (% positive) in the different groups in PC12 (**B**), Sum 185 (**D**) and Sum 159 cells (**F**) (*, †, ‡, *p* ≤ 0.05, ANOVA, *n* = 2). Only statistical comparisons between control vs. AP or Cis and Cis vs. Cis + AP are shown.

We determined if the enhanced loss of cisplatin-induced viability and increased apoptosis upon SP antagonist treatment was also accompanied by increased levels of ROS in TNBC cells. We determined the levels of cisplatin-induced ROS production in the media control group, the aprepitant alone group and in the cisplatin group in the presence and absence of aprepitant, in Sum 185 and Sum 159 TNBC cells. The levels of ROS were normalized to that of the media control group. Compared to media control, ROS levels was increased in the aprepitant treated group by 24% ± 7.2% and 38% ± 2.5%, respectively, in the Sum 185 and Sum 159 TNBC cells (Figure 4B,C; *p* < 0.05, ANOVA, *n* = 2, for both cells). The percentage increase in ROS levels in response to cisplatin was also significantly higher compared to media control (50% ± 0.7% and 21% ± 1.4%, respectively, in the Sum 185 and Sum 159 TNBC cells; Figure 3B,C; *p* < 0.05, ANOVA, *n* = 2, for both cells). The percentage increase in ROS levels in the Sum 185 cells in response to cisplatin in the absence of aprepitant was significantly lower than the cisplatin-treated cells in the presence of aprepitant (16% ± 5.1% Figure 3B; *p* < 0.05, ANOVA, *n* = 2). However, there was no significant difference in the percentage increase in ROS levels in the Sum 159 cells in response to cisplatin in the presence or absence of aprepitant (Figure 3C; *p* > 0.05, ANOVA, *n* = 2).

### 2.6. NK1R Antagonism Led to Decreased Levels of Apoptosis of PC12 Cells

We determined if NK1Rantagonist treatment could also result in the prevention of apoptosis in PC12 cells. The levels of cisplatin-induced apoptotic cells were determined in the media control group, the aprepitant alone group and in the cisplatin group in the presence and absence of aprepitant in PC12 cells by flow cytometry. The percentage of positive apoptotic cells in the media alone group was 6.64% ± 0.45%. There was no significant difference between the percentage of positive apoptotic cells in the control media group versus the aprepitant treated group (6.46% ± 1.72% (aprepitant alone) vs. 6.64% ± 0.45% (media control); Figure 4A,B; *p* ≥ 0.05, ANOVA, *n* = 2). The percentage of positive apoptotic cells was significantly higher in the cisplatin-treated group compared to media control (7.89% ± 1.04% (cisplatin alone) vs. 6.64% ± 0.45%. (media control); Figure 4A,B; *p* ≤ 0.05, ANOVA, *n* = 2). Most importantly, the percentage of positive apoptotic cells in the cisplatin-treated cells in the presence of aprepitant was significantly lower than the cisplatin-treated cells in the absence of aprepitant (6.34% ± 1.09% (cisplatin + aprepitant) vs. 7.89% ± 1.04% (cisplatin alone); Figure 4A,B; *p* ≤ 0.05, ANOVA, *n* = 2).

### 2.7. NK1R Antagonism Led to Increased Levels of Apoptosis of TNBC Cells

We determined if the enhanced loss of cisplatin-induced viability upon NK1R antagonist treatment was also accompanied by increased levels of apoptosis in TNBC cells. We determined the levels of cisplatin-induced apoptotic cells in the media control group, the aprepitant alone group and in the cisplatin group in the presence and absence of aprepitant in Sum 185 and Sum 159 TNBC cells. The percentage of positive apoptotic cells in the media alone group was 5.26% ± 0.15% and 8.69% ± 0.13%, respectively, for the Sum 185 and Sum 159 cells. Compared to media control, the percentage of positive apoptotic cells was significantly higher in the aprepitant treated group (8.21% ± 0.69% (aprepitant alone) vs. 5.26% ± 0.15% (media control) and 13% ± 0.84% (aprepitant alone) vs. 8.69% ± 0.13% (media control)), respectively, for the Sum 185 and Sum 159 cells (Figure 4C–F; *p* ≤ 0.05, ANOVA, *n* = 2, for both cells). The percentage of positive apoptotic cells was also significantly higher in the cisplatin-treated group compared to media control (9.41% ± 0.56% (cisplatin alone) vs. 5.26% ± 0.15% (media control) and 13.35% ± 0.17% (cisplatin alone) vs. 8.69% ± 0.13% (media control), respectively, for the Sum 185 and Sum 159 cells, (Figure 4C–F; *p* ≤ 0.05, ANOVA, *n* = 2, for both cells). Most importantly, the percentage of positive apoptotic cells in the cisplatin-treated cells in the presence of aprepitant was significantly higher than the cisplatin-treated cells in the absence of aprepitant (12.7% ± 0.14% (cisplatin + aprepitant) vs. 9.41% ± 0.56% (cisplatin alone) and 14.8% ± 0.21% (cisplatin + aprepitant) vs. 13.35% ± 0.17% (cisplatin alone), respectively, for the Sum 185 and Sum 159 cells. (Figure 4C–F; *p* ≤ 0.05, ANOVA, *n* = 2, both cells).

### 2.8. Effects of NK1R Antagonism on the Transcriptome in TNBC Cells

In order to determine the mechanisms by which NK1R antagonism increased the efficacy of cisplatin. We determined levels of mRNA by RNA-Seq in Sum 185 TNBC cells that were treated with and without cisplatin in the presence and absence of aprepitant.

**Figure 5:**
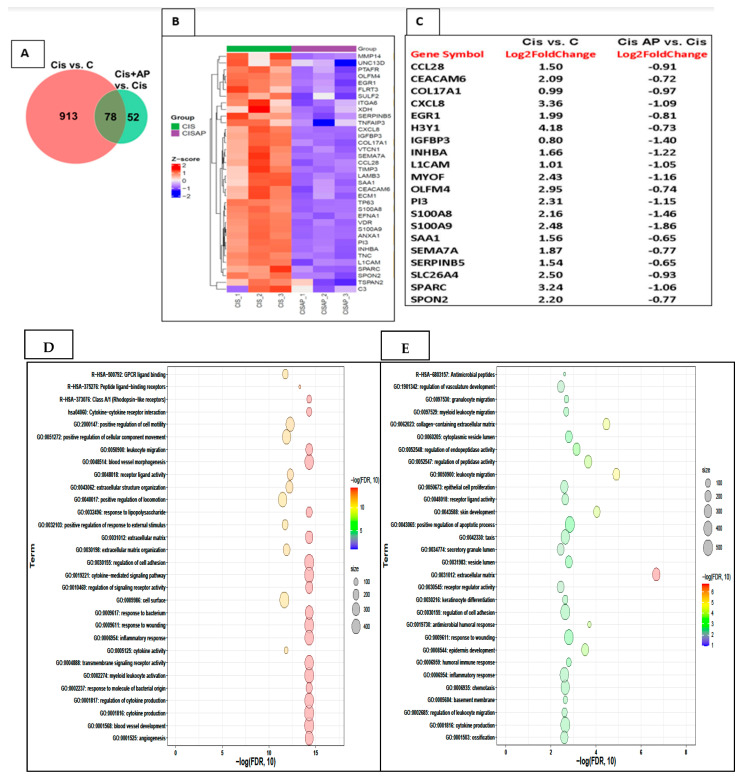
Effects of NK1R antagonism on the transcriptome in Sum 185 TNBC cells. (**A**) Venn diagram showing differentially expressed genes between 2 comparisons CIS vs. C and CIS + AP vs. CIS (**B**) Heat map showing the upregulation (Z-score scaled) of genes linked to pro-inflammatory responses, extracellular matrix (ECM) and cell adhesion in CIS vs. CIS + AP. (**C**) Top 20 differentially expressed genes (**D**,**E**) Functional enrichment analysis of the upregulated gene sets in CIS and downregulated gene sets in CIS + AP using Gene Ontology (GO) and Kyoto Encyclopedia of Genes and Genomes (KEGG) annotations showing top 30 enriched pathways.

To identify candidate mRNAs that contributed to the increased efficacy of cisplatin, we performed two comparisons: CIS vs. no CIS mRNA sets and CIS + AP vs. CIS mRNA sets. In the CIS vs. no CIS comparison, the total number of genes determined was 991, of which 612 genes were upregulated and 379 genes were down-regulated. In the CIS + AP vs. CIS comparison, the total number of genes determined was 130, of which 44 genes were upregulated and 86 genes were down-regulated. We further identified genes that were differentially expressed between the two comparisons: CIS vs. No CIS mRNA sets and CIS + AP vs. CIS mRNA sets. We determined differentially expressed genes with 1.5-fold change and false discovery rate (FDR) < 0.05 (*n* = 3). There were 78 genes that were differentially expressed between the two comparisons (Figure 5A). Of the 78 genes, 68 genes were upregulated with CIS but downregulated in CISAP, and 10 genes were downregulated with CIS but upregulated in CISAP.

Out of the 68 genes that were upregulated with CIS but downregulated in CISAP, 36 genes represented in the heat map in Figure 5B–E are genes associated with pathways linked to pro-inflammatory responses, extracellular matrix (ECM) and cell adhesion such as *MMP14*, *UNC13D*, *PTAFR*, *OLFM4*, *EGR1*, *FLRT3*, *SULF2*, *ITGA6*, *XDH*, *SERPINB5*, *TNFAIP3*, *CXCL8*, *IGFBP3*, *COL17A1*, *VTCN1*, *SEMA7A*, *CCL28*, *TIMP3*, *LAMB3*, *SAA1*, *CEACAM6*, *ECM1*, *TP63*, *S100A8*, *EFNA1*, *VDR*, *S100A9*, *ANXA1*, *PI3*, *INHBA*, *TNC*, *L1CAM*, *SPARC*, *SPON2*, *TSPAN2* and *C3*.

Ten genes were downregulated with CIS but upregulated in CISAP; importantly, out of the 10 genes, only one gene, Stearoyl-CoA desaturase (*SCD*), was increased more than two-fold change. *SCD* is one of the genes that is involved in ameliorating the neuropathic phenotype induced by diabetes by restoring aberrant fatty acid biosynthesis and thereby preventing altered myelin lipid profile and ensuing myelin structural abnormalities [25]. Of special relevance is that two genes, although increased by only 1.5-fold, are genes known to be associated with better prognosis in TNBC (*PTPRD* and *Neurl1*). *PTPRD* is associated with negative regulation of stemness, epithelial-mesenchymal transition (EMT), and migration and invasion in breast cancer cells [26].

*Neurl1* overexpression is known to downregulate Notch signaling, which is a key pathway associated with tumor growth, metastases and chemoresistance [27].

## 3. Material and Methods

### 3.1. Cell Culture

Rat neuronal cell lines, PC12, were purchased from the American Type Culture Collection (Manassas, VA, USA). Human TNBC cell lines, SUM 159 and SUM 185, were a kind courtesy of Dr. Nato Ueno, Translational Breast Cancer Research, Department of Breast Medical Oncology, MD Anderson Cancer Center, Houston, TX, USA. The SUM 159 and SUM 185 were cultured in RPMI containing 10% heat-inactivated fetal bovine serum, antibiotics (streptomycin and penicillin), an antifungal agent (amphotericin B; all from Invitrogen, Carlsbad, CA, USA) and were not passaged more than 4 weeks continuously. PC12 cells were cultured in the same media used for SUM 185 and SUM 159 cells but also contained 10% heat-inactivated horse serum and 100 ng/mL nerve growth factor (NGF). Cisplatin and aprepitant (respectively, Cat nos. S1166 and S1189) were purchased from Selleck-Chemicals, Houston, TX, USA.

### 3.2. Viability Assay

In order to determine the effect of aprepitant on the cellular metabolic activity as an indicator of cell viability, proliferation and cytotoxicity cell viability, we used the MTT assay. We plated in a 96-well plate (3000 cells/well; SUM 159, SUM 185 or PC12 cells per well); after 24 h, we treated triplicate wells with cisplatin (0.001 µm to 100 µm) in the presence or absence of10 µm of AP (2 h before cisplatin treatment and continuing until termination). We selected 10 µm of AP based on it being the IC_10_ of Sum 185 and Sum 159, and for consistency purposes, we used 10 µm for all cells. Control wells were treated with media or AP alone. Following 48 h treatment with cisplatin with or without AP, we determined the cell viability. Each well was emptied, and MTT (1 mg/mL in medium containing 1% serum) was then added to each well and incubated at 37 °C for 2 h. Following which viable cells that contain NADPH-dependent oxidoreductase enzymes reduce the MTT to formazan. The insoluble formazan crystals were solubilized in an extraction buffer containing 20% sodium dodecyl sulfate and 50% dimethylformamide. The cells were incubated overnight with the extraction buffer at 37 °C, following which the optical density was measured at 590 nm using a 96-well multi scanner (Molecular Devices, Sunnyvale, CA, USA). Data are represented as percentage viability related to untreated cells ± SEM.

### 3.3. ROS Measurement

ROS levels were determined by the dichlorofluorescein diacetate (DCFDA) assay kit (Cat no. C6827, Thermofisher Scientific, Waltham, MA, USA). We treated SUM 159, SUM 185 or PC12 cells with each of their respective IC50 concentrations of cisplatin (as deduced from the MTT experiments) with or without AP (10 µm, 2 h before cisplatin treatment and continuing until termination (48 h)). Controls consisted of treatment with media or AP alone. Following incubation with each of the conditions, the wells were emptied and washed once with 1× PBS, following which each of the cells was treated with CM-H2DCFDA (10 μM in 1× PBS) for 30 min at 37 °C in the dark. The wells were then read using a fluorescence spectrophotometer with maximum excitation and emission spectra of 495 nm and 517 nm, respectively. Data are represented as fluorescence intensity ± SEM for each group.

### 3.4. Flow Cytometry

To determine the NK1R expression, Sum 185, Sum 159 or PC12 cells cultured with cisplatin were washed with their respective complete media and stained with LIVE/DEAD Zombie Aqua™ (423101, BioLegend^®^, San Diego, CA, USA and NK1R APC/Cy7 (NB300-119APCCY7, Novus Biologicals^®^, (Littleton, CO, USA). To determine apoptosis of the cells, Annexin V/PI staining was performed using a commercially available kit (640914, BioLegend^®^). Briefly, after culture, the cells were washed once with the media and once with FACS buffer then suspended in Annexin V Binding Buffer at a concentration of 0.25–1.0 × 10^7^ cells/mL. One hundred microliters of cell suspension was stained with 5 μL of FITC Annexin V and 10 μL of Propidium Iodide Solution, gently vortexed, and incubated for 15 min at room temperature (25 °C) in the dark. Subsequently, 400 μL of Annexin V Binding Buffer was added to each tube prior to the acquisition. The samples were acquired by CytoFlex™ (Indianapolis, IN, USA) and analyzed with FlowJo software version 10.7.1 (Ashland, OR, USA).

### 3.5. RNA Isolation and Library Preparation and RNA-Seq Data Analysis

We isolated RNA from SUM 185 cells following 48 h treatment with cisplatin in the presence and absence of 10 µm AP, and controls included media alone with and without AP. For the RNA-Seq experiments, as previously [28], RNA was briefly extracted with a Purelink Kit (Ambion, Life Technologies, Carlsbad, CA, USA) following reverse-transcription to cDNA using SuperScript III Reverse Transcriptase (Invitrogen, Waltham, MA, USA). We then performed enrichment of Poly (A)-tailed mRNA and prepared the RNA-seq library using the UT Cancer Genomics Core Center facility following manufacturer’s instructions outlined in the KAPA mRNA HyperPrep Kit (KK8581, Roche, Holding AG, city, Switzerland) and KAPA Unique Dual-indexed Adapter kit (KK8727, Roche). We then performed RNA-seq using the Illumina Nextseq550 (San Diego, CA, USA) with the 75 bp pair ended running mode.

Cutadapt v1.15 was used to preprocess the raw mRNA sequence reads to remove bases with quality scores < 20 and adapter sequences. The clean RNA-seq reads were then aligned to human genome assembly GRCh38 with STAR v2.5.3a. HTseq-count with default parameter using annotation from ENSEMBL v102 was used to count uniquely mapped reads overlapping genes. Only genes with >5 reads in at least one sample were retained. We then performed differential expression analysis only on the raw read counts of retained genes using DESeq2 software, which uses a model based on the negative binomial distribution [29]. Benjamini and Hochberg’s approach was used to attain *p*-values adjusted to control for false discovery rate (FDR). Genes with fold change (FC) > 1.5 and FDR < 0.05 were assigned as differentially expressed genes (DEGs). A standard gene set enrichment analysis was performed with a hypergeometric test using WebGestalt v 0.4.3 [30]. The resulting *p* values were also adjusted using Benjamini and Hochberg’s approach.

### 3.6. Statistical Analyses

Data represented are mean ± SEM of a minimum of two experiments. We determined statistical differences using analysis of variance (ANOVA), followed by Tukey’s or Dunn’s posttest as appropriate or by Student’s unpaired *t*-test. The threshold of statistical significance was set at *p* ≤ 0.05. We used the Graph Pad Prism version 6.04 for Windows, Graph Pad Software (San Diego, CA, USA) for performing data and statistical analysis.

## 4. Discussion

Though cisplatin is very effective as a treatment strategy in TNBC, it has unwarranted outcomes owing to recurrence, chemoresistance and detrimental side effects. There is an urgent need for effective and safe therapeutics for treating TNBC.

We determined if NK1R antagonism in combination with cisplatin may serve as a novel, more efficacious and safer therapeutic option than existing therapies for TNBC.

We treated a neuronal cell line (PC12) and two TNBC cell lines (Sum 185 and Sum 159) with aprepitant, an NK1R antagonist that is widely used to attenuate chemotherapy-associated nausea, and demonstrated the following responses. We demonstrated that (a) aprepitant decreased cisplatin-induced loss of viability, ROS production and apoptotic cell death in PC12 cells compared with cells treated with cisplatin alone and (b) aprepitant increased cisplatin-induced loss of viability, ROS production and apoptotic cell death in TNBC cells compared with cells treated with cisplatin alone.

The current studies are extremely important and timely. Treatment of TNBC is extremely challenging compared to other breast cancers that express one or more receptors such as the progesterone receptor (PR), estrogen receptor (ER) and human epidermal growth factor receptor (HER2). The expression of these receptors makes it more amenable for treatment with chemotherapeutic agents that block these receptors. TNBC cells lack these three receptors, resulting in limited treatment options [2]. Although cisplatin is very effective as a treatment strategy in TNBC, it can cause challenging side effects such as nerve damage, kidney damage, hearing loss and other manifestations [9,31]. Furthermore, recurrence and chemoresistance are commonly noted in TNBC patients treated with cisplatin. Our strategy to use NK1R antagonism in combination with cisplatin is aimed at targeting multiple pathways that are associated with TNBC growth, metastases and development of chemoresistance. Our strategy has importantly resulted in using lesser amounts of cisplatin to achieve tumor cytotoxicity, thus probably serving as an important mechanism to attenuate the side effects of cisplatin. Our studies demonstrating the ability of SP receptor antagonism to enhance the efficacy of cisplatin in TNBC cells and at the same time protect neuronal cells from cisplatin-induced toxicity addresses the challenges outlined above.

The mechanism by which cisplatin, a DNA intercalating agent, induces its anti-tumor effects is via crosslinking DNA with resultant interference with RNA transcription and DNA replication leading to cell-cycle arrest and apoptosis in tumor cells [31]. There are several mechanisms by which tumor cells attain chemoresistance to cisplatin, such as decreased levels of cisplatin being accumulated in tumor cells as a result of (a) lesser uptake or enhanced efflux of the drug, (b) detoxification of the drug by intrinsic mechanisms mediated by tumor cells, (c) enhanced repair of the DNA or (d) negative regulation of apoptotic mechanisms [31,32].

Besides the ability of cisplatin to intervene with the proliferation of tumor cells as a result of the formation of deoxyribonucleic acid (DNA)-platinum adducts, it mediates its effects on tumor cells by causing elevated production of reactive oxygen species leading to alteration of mitochondrial function and activation of apoptotic pathways [33]. We demonstrated that SP receptor antagonism enhanced the cisplatin-induced ROS production and apoptosis levels in two TNBC cancer cells. Importantly, our RNA-Seq studies determined that SP receptor antagonism attenuates the levels of several genes associated with apoptosis, such as *OLFM4*, *CCL28*, and *TNFAIP3* (anti-apoptotic) [34,35].

Furthermore, we demonstrated that important genes associated with metastases are attenuated with SP receptor antagonist treatment. One of the main mechanisms by which metastases occur is via the ability of cancer cells to degrade basement membranes and spread to other tissues via blood or lymphatic vessels [36]. *MMP14*, a known mediator of extracellular matrix (ECM) degradation and ensuing metastases [37,38,39], is attenuated with SP receptor antagonist treatment. Other metastases genes associated with poor prognosis in breast cancer, such as *CEACAM6*, *COL17A1*, *CXCL8*, *TNFAIP3*, *SEMA7A* and *L1CAM* [31,38,39,40,41], are also attenuated with SP receptor antagonist treatment.

Studies have shown that inflammation is associated with the aggressive phenotype of TNBC [42,43]. We determined that SP receptor antagonism attenuates the levels of genes associated with inflammatory processes such as *S100A8*, *S100A9*, *CXCL8*, *CCL28*, *ANXA1* and *SAA1* [42,44,45,46,47].

Furthermore, genes associated with recurrence and chemoresistance that are attenuated by SP receptor antagonism include *IGFBP-3*, *PI3* and *SPARC* [48,49,50]. Moreover, *Histone H3Y1*, an important gene that positively controls cell cycle progression and cell growth, is attenuated by SP receptor antagonism [51].

Genes that are attenuated with SP receptor antagon.ism, such as *SERPINB5*, are known to have a controversial role in TNBC. Studies show that the absence of *SERPINB5* (Maspin) is an indicator of tumor progression and metastatic potential, while other studies show that Maspin expression correlates with an aggressive phenotype in breast cancer and with poor prognosis. The outcome varied with subcellular Maspin expression, and nuclear staining was demonstrated to be significantly associated with a better prognosis than cytoplasmic staining [52,53].

Genes that are attenuated with SP receptor antagonism, such as *SPON2,* are involved in cancer progression and metastasis of many tumors other than breast cancer [54]. Similarly, *INHBA* was used as a diagnostic marker in ovarian cancer [55], but there are no published studies that have determined its expression and/or role in breast cancer. The only gene that was downregulated by SP receptor antagonism but was beneficial in TNBC was *EGR1*, which is a tumor suppressor gene [56].

Cisplatin is one of the chemotherapeutic agents that causes chemotherapy-induced peripheral neuropathy (CIPN). CIPN often leads to the manifestation of ongoing pain as a result of damage to peripheral sensory and motor neurons in cancer patients that were treated with platinum-based chemotherapeutic agents such as cisplatin and oxaliplatin [31]. CIPN occurs in a dose- and time-dependent manner [57]. The initial symptoms of the onset of CIPN can vary from its occurrence as early as after the first dose or after several cycles of therapy [58,59]. One of the mechanisms leading to cisplatin-induced neuropathy is due to oxidative stress, mitochondrial dysfunction and induction of apoptosis [33]. PC12 cells have been routinely used to study cisplatin-induced neurotoxicity [60]. Our findings revealed that aprepitant attenuated cisplatin-induced neurotoxicity through inhibition of ROS-production and apoptosis. Our studies demonstrating the protective effect of aprepitant in PC12 neuronal cells are concurrent with other studies wherein aprepitant has been demonstrated to protect against cisplatin-induced nephrotoxicity and hepatotoxicity [61].

In the current studies, we determined that the levels of NK1R were significantly elevated in response to cisplatin in a rat neuronal cell line and in two TNBC cell lines. We also determined that the levels of ROS and apoptosis were significantly increased in response to cisplatin treatment. We determined that treatment with aprepitant (an NK1R antagonist that is widely used to attenuate chemotherapy-associated nausea) reversed one or more of these cisplatin-induced alterations in the rat neuronal cell line and in TNBC cells. Most importantly, we determined that aprepitant enhance the efficacy of cisplatin in TNBC cells while protecting PC12 cells from cisplatin-induced oxidative stress and apoptosis. The studies in this manuscript will determine if SP receptor antagonism in combination with cisplatin may serve as a novel, more efficacious and safer therapeutic option than existing therapies for TNBC.

## 5. Conclusions

These studies, if proven in animal models of chemotherapy-induced peripheral neuropathy (CIPN), could lead to the possibility of using SP antagonism as a therapeutic intervention to prevent chemotherapy-induced neurotoxicity. Most importantly, cisplatin is known to be highly effective in several cancers with increased survival rates. However, the side effects of neurotoxicity and ensuing CIPN often lead to the necessity for dose de-escalation, pausing of therapy or replacement with less effective chemotherapeutic regimens. These studies pursuant to animal studies could possibly determine the use of NK1R antagonism as a novel therapeutic strategy for the prevention of cisplatin-induced neurotoxicity and enhancement of the efficacy of chemotherapy in cancer.

## Data Availability

We are in the process of depositing our RNA-Seq data into Geo database, which can then be accessed by entering the manuscript information.
