# Erratum: Rodriguez et al. Substance P Antagonism as a Novel Therapeutic Option to Enhance Efficacy of Cisplatin in Triple Negative Breast Cancer and Protect PC12 Cells against Cisplatin-Induced Oxidative Stress and Apoptosis. *Cancers* 2021, *13,* 3871

**DOI:** 10.3390/cancers13205178

**Published:** 2021-10-15

**Authors:** Emma Rodriguez, Guangsheng Pei, Zhongming Zhao, Sang T. Kim, Alexis German, Prema Robinson

**Affiliations:** 1 Department of Infectious Diseases, Infection Control and Employee Health, The University of Texas MD Anderson Cancer Center, Houston, TX 77030, USA; EERodriguez1@mdanderson.org; 2 Center for Precision Health, School of Biomedical Informatics, The University of Texas Health Science Center at Houston, Houston, TX 77030, USA; guansheng.pei@uth.tmc.edu (G.P.); Zhongming.Zhao@uth.tmc.edu (Z.Z.); 3 Department of General Internal Medicine, Division of Internal Medicine, The University of Texas MD Anderson Cancer Center, Houston, TX 77030, USA; STKim@mdanderson.org; 4 University of Houston, Houston, TX 77004, USA; abgerman@uh.edu

One contributor’s name was missing in the original version of the authorship of the paper [1]. This author is Zhongming Zhao, who should be listed as the third author. He belongs to the original affiliation 2 (Zhongming Zhao ^2^):

^2^ Center for Precision Health, School of Biomedical Informatics, The University of Texas Health Science Center at Houston, Houston, TX 77030, USA

The updated authorship is listed as below:

Emma Rodriguez, Guangsheng Pei, Zhongming Zhao, Sang T. Kim, Alexis German and Prema Robinson

The “Author Contributions” statement thus should be updated to the following version:

**Author Contributions:** Conceptualization, P.R.; methodology, P.R., E.R., S.T.K., G.P., and Z.Z.; formal analyses, P.R., S.T.K., G.P., and Z.Z.; investigation, A.G.; original draft preparation, P.R.; review and editing, P.R., E.R., S.T.K., and G.P. All authors have read and agreed to the published version of the manuscript.

The authors have confirmed that the updated authorship meets the ICMJE criteria, the guidelines of which are followed by this journal. We apologize for this error and state that the scientific conclusions are unaffected. The original article has been updated.
